# IL-34 and CSF-1 display an equivalent macrophage differentiation ability but a different polarization potential

**DOI:** 10.1038/s41598-017-18433-4

**Published:** 2018-01-10

**Authors:** Sonia Boulakirba, Anja Pfeifer, Rana Mhaidly, Sandrine Obba, Michael Goulard, Thomas Schmitt, Paul Chaintreuil, Anne Calleja, Nathan Furstoss, François Orange, Sandra Lacas-Gervais, Laurent Boyer, Sandrine Marchetti, Els Verhoeyen, Frederic Luciano, Guillaume Robert, Patrick Auberger, Arnaud Jacquel

**Affiliations:** 1 Université Côte d’Azur, C3M Inserm, U1065 Nice, France; 2 Université Côte d’Azur, Centre Commun de Microscopie Appliquée, Nice, France

## Abstract

CSF-1 and IL-34 share the CSF-1 receptor and no differences have been reported in the signaling pathways triggered by both ligands in human monocytes. IL-34 promotes the differentiation and survival of monocytes, macrophages and osteoclasts, as CSF-1 does. However, IL-34 binds other receptors, suggesting that differences exist in the effect of both cytokines. In the present study, we compared the differentiation and polarization abilities of human primary monocytes in response to CSF-1 or IL-34. CSF-1R engagement by one or the other ligands leads to AKT and caspase activation and autophagy induction through expression and activation of AMPK and ULK1. As no differences were detected on monocyte differentiation, we investigated the effect of CSF-1 and IL-34 on macrophage polarization into the M1 or M2 phenotype. We highlighted a striking increase in IL-10 and CCL17 secretion in M1 and M2 macrophages derived from IL-34 stimulated monocytes, respectively, compared to CSF-1 stimulated monocytes. Variations in the secretome induced by CSF-1 or IL-34 may account for their different ability to polarize naïve T cells into Th1 cells. In conclusion, our findings indicate that CSF-1 and IL-34 exhibit the same ability to induce human monocyte differentiation but may have a different ability to polarize macrophages.

## Introduction

Monocytes are circulating blood leukocytes that play important role in tissue homeostasis and in the inflammatory response which is essential for the innate response to pathogens^1^. They have the unique property among peripheral blood cells to migrate into tissues where they are subjected to differentiation into morphological and functionally heterogeneous cells that include macrophages, myeloid dendritic cells, and osteoclasts, depending on the stimulus^2^. The differentiation of peripheral blood monocytes into resident tissue macrophages can be recapitulated *ex vivo* by incubation in the presence of colony-stimulating factor-1 (CSF-1)^3^. The biologic effects of CSF-1 are mediated by a unique receptor, the CSF-1R, which is encoded by the *c-fms* proto-oncogene^4^. Downstream signaling pathways activated by CSF-1R upon ligand binding include PI3K-AKT and AMPK pathways, which are implicated in the respective activation of caspases and autophagy, two key processes required for CSF-1-induced macrophage differentiation^5^. Our previous studies have established that physiological monocyte differentiation triggered by CSF-1R engagement is critically dependent on the oscillatory activation of the kinase AKT, which within 2–3 days leads to the formation of a multi-molecular platform that includes the adaptor Fas-associated death domain (FADD), the serine-threonine kinase RIP1, the long and short isoforms of FLIP, and procaspase-8^6^. Caspase-8 activation in this platform provokes a limited activation of several downstream caspases that cleave specific intracellular proteins^7,8^. The contribution of these cleavages to the CSF-1–driven monocyte-to-macrophage differentiation remains poorly understood. More recently, we have also established that autophagy plays a crucial role during macrophage differentiation of monocytes^9^. We found that CSF-1 increases the expression of the purinergic receptor P2RY6 that in turn activates the CAMKK2-AMPK-ULK1 pathway leading to autophagy induction^10^. Notably, inhibition of this pathway abrogated both CSF-1-mediated induction of autophagy and differentiation.

IL-34 is a newly identified cytokine with only partially understood functions. IL-34 has been recently identified as the second ligand for CSF-1R through comprehensive proteomic analyses^11^. Although it lacks appreciable similarity with CSF-1 or any other proteins, IL-34 tightly binds to CSF-1R and promotes the differentiation, proliferation and survival of monocytes, macrophages and osteoclasts as CSF-1 does^12,13^. IL-34 actions have been rendered more complex by the discovery of two other distinct receptors: the receptor type protein-tyrosine phosphatase zeta (PTP-ζ)^14^ and CD138 (Syndecan 1)^15^, suggesting additional roles for IL-34, compared to CSF-1. Recently, IL-34 was found to be associated with the inflammation process seen in diseases such as rheumatoid arthritis (RA)^16^, inflammatory bowel disease^17^ and Sjogren’s syndrome^18^. Interestingly, emerging findings indicate that IL-34 levels are abnormally increased in serum and synovial fluid in patients with reactive rheumatoid arthritis^19,20^.

Here, we used human primary monocytes to characterize in details IL-34 and CSF-1-driven macrophage differentiation and polarization into the M1 or M2 phenotype. We demonstrate that IL-34, like CSF-1, induced caspase and autophagy activation and that both processes are required to induce differentiation of monocytes into macrophages in response to IL-34. Moreover, we report that IL-34 and CSF-1 macrophages display a different polarization potential when polarized by pro or anti-inflammatory stimulus, since we found some differences in the mRNA and protein expression profiles of some cytokines/chemokines in CSF-1 and IL-34 differentiated cells polarized into the M1 or M2 phenotype.

## Results

### CSF-1 and lL-34 exhibit similar signaling and differentiation properties in primary human monocytes

**Figure 1:**
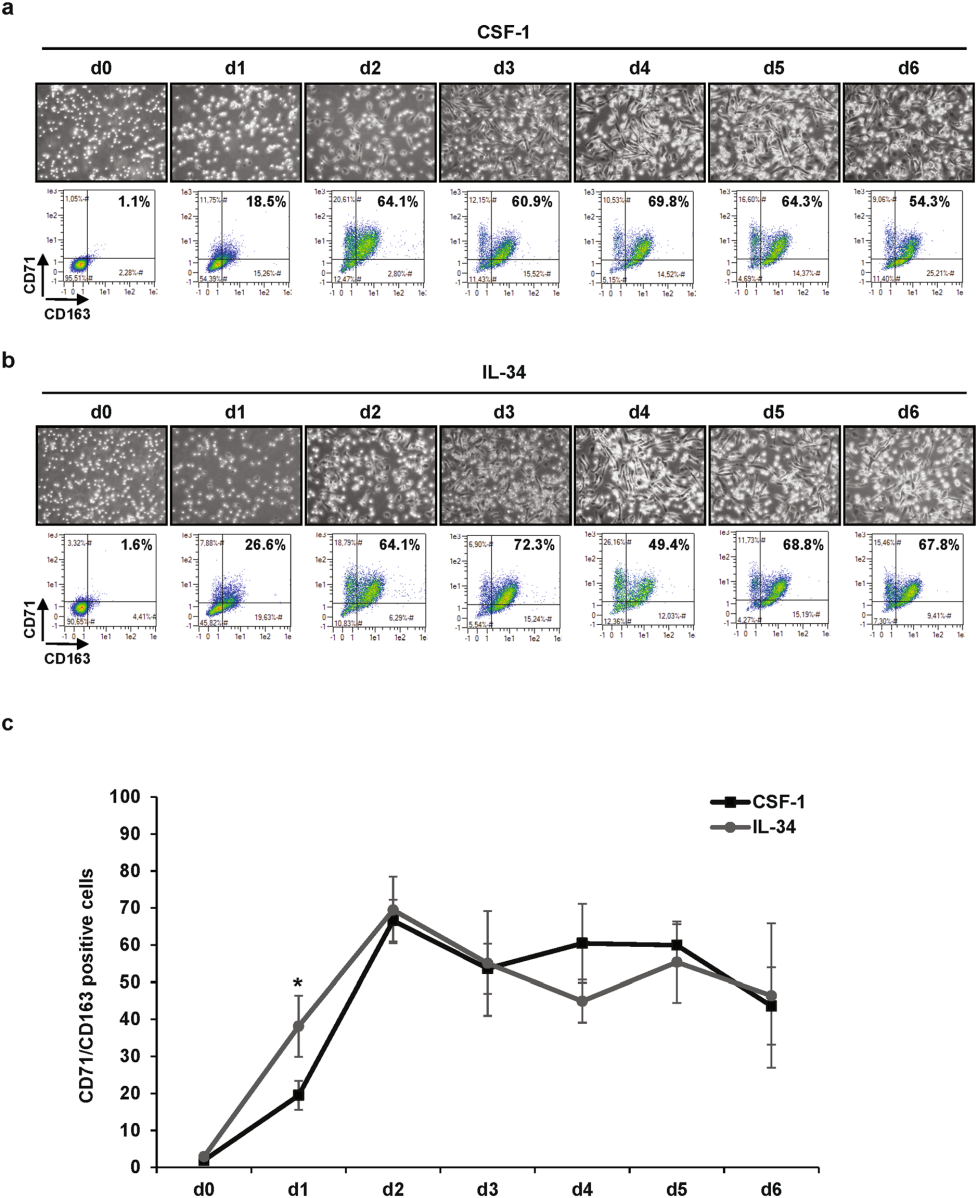
IL-34 and CSF-1 induce equivalent macrophagic differentiation of primary monocytes. Human peripheral blood monocytes from healthy donors were exposed to 100 ng/mL CSF-1 or 100 ng/mL IL-34 for the indicated times. (**a**,**b**) Macrophage differentiation was examined morphologically (fibroblastic shape) and by 2-color flow cytometric analysis. The percentage indicates cells that express both CD71 and CD163. (**c**) Macrophagic differentiation of monocytes from 3 different healthy donors was followed by 2-color flow cytometric analysis. The percentage indicates cells that express both CD71 and CD163. *P < 0.05 according to a paired student *t* test.

When stimulated with 100 ng/mL CSF-1, human monocytes differentiate into macrophages as shown by an increase in cell adherence and the acquisition of specific markers such as TFRC/CD71 and CD163. At day 2, 60–70% of myeloid cells were found to be positive for both CD71 and CD163 expression (Fig. 1a). This high rate of differentiation was maintained up to 6 days. The kinetic of macrophage differentiation induced by 100 ng/mL lL-34, the second known ligand of the CSF-1R, was nearly indistinguishable from that of CSF-1 (Fig. 1b and c, Sup Fig. 1a and b). As CSF-1 and IL-34 bind the CSF-1R with differential affinities, we performed a dose-response curve for each cytokine on monocyte differentiation (Sup Fig. 1c). We confirmed that for each cytokine, a 100 ng/mL concentration triggers maximal differentiation capacity.

**Figure 2:**
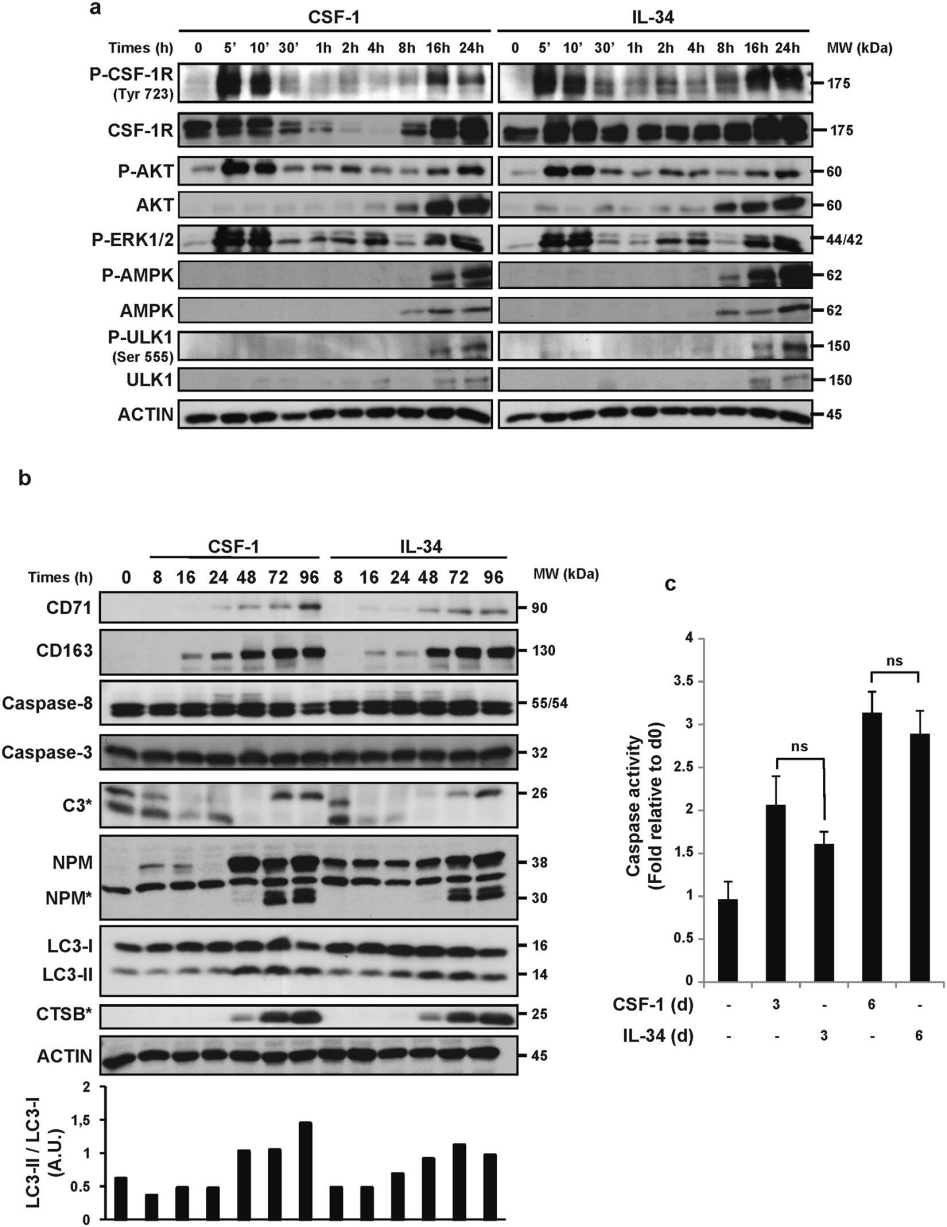
Caspases and autophagy are activated upon IL-34 or CSF-1 treatment. Human peripheral blood monocytes from healthy donors were exposed to 100 ng/mL CSF-1 or 100 ng/mL IL-34 for the indicated times. (**a**) Immunoblot analysis of indicated proteins in monocytes following CSF-1 or IL-34 stimulation. P indicate phosphorylated proteins. Each panel is representative of at least 3 independent experiments. (**b**) Immunoblot analysis of indicated proteins in monocytes following CSF-1 or IL-34 stimulation. The ratio of the LC3-II protein level to that of LC3-I protein level was determined using ImageJ software. Actin was detected as the loading control. Asterisks indicate cleavage fragments. Each panel is representative of at least 3 independent experiments. (**c**) Caspase activity was quantified by flow cytometry analysis using DEVD-FITC. The results are expressed as the fold induction compared with untreated cells and represent the mean ± SD of 3 independent experiments performed in duplicate. n.s. denotes non-significant according to a paired student *t* test.

We next analyzed the early signaling pathways associated with the engagement of the CSF-1 receptor by either CSF-1 or lL-34 in human primary monocytes. We show that both CSF-1 and lL-34 triggered a transient first wave of CSF-1R phosphorylation at 5 and 10 min and a second one at 16 and 24 h (Fig. 2a). For both cytokines, the second wave of phosphorylation was associated with an increase in CSF-1R protein expression. The same waves of phosphorylation and activation of AKT and ERK 1/2 were also observed in CSF-1 and lL-34-stimulated monocytes. We have previously shown that CSF-1 triggered long term phosphorylation and activation of AMPK and ULK1 that correlated with increased autophagy in primary human monocytes^10^. This increased phosphorylation and activation of AMPK and ULK1 also correlated with an enhanced expression of both AMPK and ULK1 proteins (Fig. 2a). CSF-1-mediated phosphorylation and activation of AMPK and ULK1 were confirmed in the present study, but we further established for the first time that lL-34 increased AMPK and ULK1 phosphorylation at 16 and 24 h. This activation of phosphorylation was accompanied with increased expression of AMPK and ULK1 (Fig. 2a). In addition, we also investigated the effect of increasing concentrations of both cytokines on the early signaling events triggered by CSF-1R engagement and we confirmed a slightly higher sensitivity to CSF-1 (Sup Fig. 2a, please see also Sup Fig. 1c). Nevertheless, a 100 ng/mL concentration was maximal for both cytokines. We next compared the ability of CSF-1 and lL-34 to promote differentiation of monocytes. Both cytokines equivalently increased the protein expression of CD71 and CD163, two macrophage differentiation markers at 48 h (Fig. 2b). CSF-1 and IL-34 induced the activation of both caspases 8 and 3, which are a prerequisite for monocyte differentiation into macrophage and for NPM cleavage (Fig. 2b)^21^. We also analyzed the lipidation and cleavage of LC3-l into LC3-ll, a hallmark of autophagy induction in monocytes stimulated with CSF-1 or lL-34. Autophagy induction, that was increased after 48 h of stimulation, correlated with Cathepsin B (CTSB) activation in both CSF-1 and lL-34 treated monocytes (Fig. 2b)^9^. ln addition, caspase-3 activity at 3 and 6 days were found to be equivalent in CSF-1 or lL-34-treated monocytes (Fig. 2c). Furthermore, electron microscopy images of CSF-1 or lL-34 differentiated monocytes at day 3 showed accumulation of autolysosomes (Sup Fig. 2b), CTBS active fragment and increase in autophagic flux in the presence of Bafilomycin A1 (Sup Fig. 2c).

**Figure 3:**
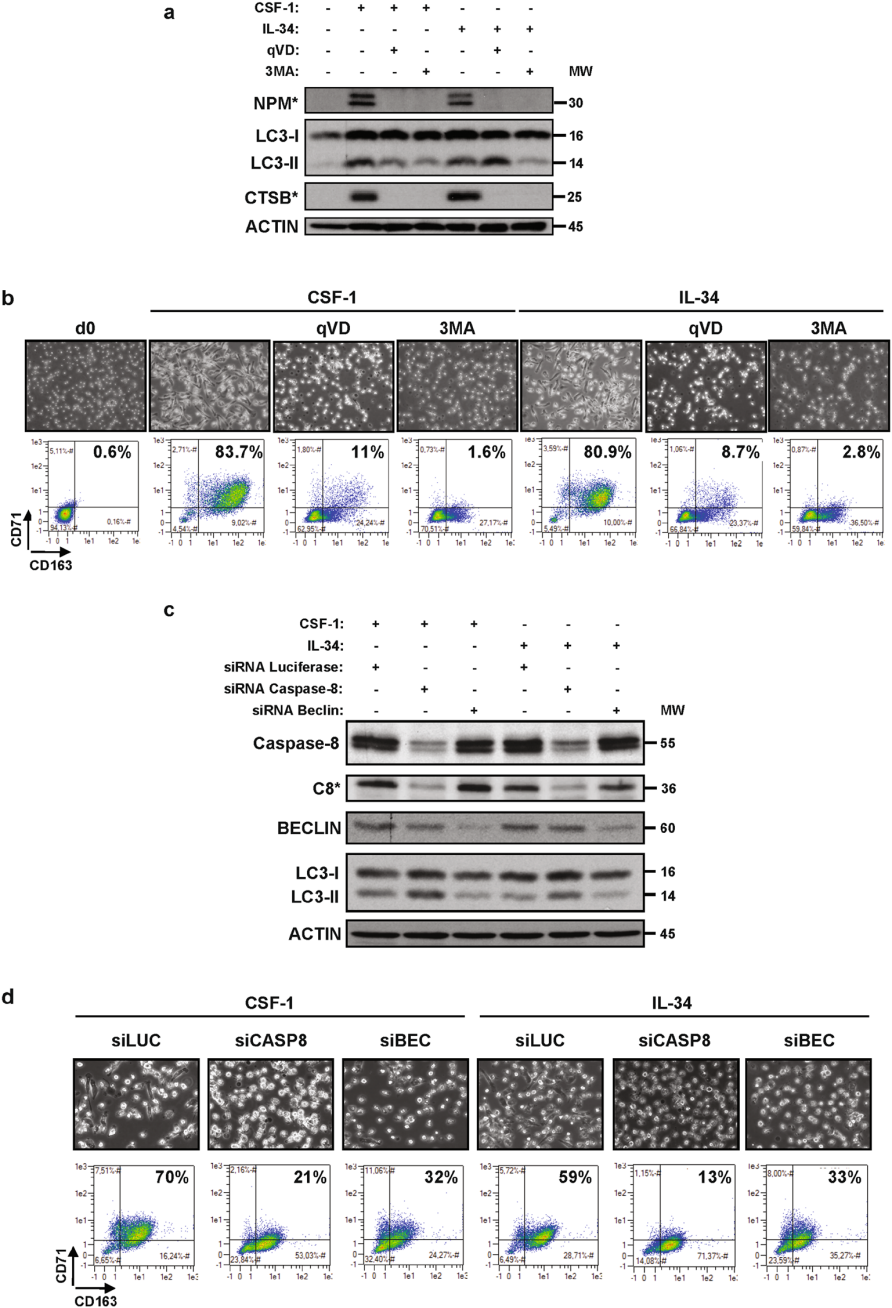
Caspases and autophagy are required for CSF-1 or IL-34 induced macrophagic differentiation. (**a**) Human monocytes were exposed for 2 days to 100 ng/mL CSF-1 or 100 ng/mL IL-34 alone or in combination with either 50 µM qVD or 3 mM 3MA (3-MethylAdenine), which were added 30 min before CSF-1 or IL-34 treatment. The expression of cleaved NPM (NPM*), LC3B and cleaved cathepsin B (CTSB*) was analyzed by immunoblotting. (**b**) Human monocytes were treated as in Fig. 3a. (**c**,**d**) Monocytes were transfected with siRNA targeting *LUCIFERASE* (LUC), *CASPASE-8* (CASP8) or *BECLIN* (BEC) and exposed for 2 days to 100 ng/mL CSF-1 or 100 ng/mL IL-34. (**c**) Expression of Caspase-8, Beclin and LC3B was analyzed by immunoblotting. Actin is used as a loading control. Asterisk indicates a cleavage fragment. (**d**) Differentiation was examined as previously described.

Finally, CSF-1 and lL-34 both triggered NPM cleavage, LC3-ll accumulation and CTSB activation at 2 days (Fig. 3a). Caspases or autophagy inhibition by qVD or 3MA, respectively, abolished NPM and LC3-l cleavage, CTSB activation and macrophage differentiation (Fig. 3a and b). Knock-down of caspase 8 was found to increase CSF-1 and lL-34-mediated LC3-ll accumulation while knock-down of BECLIN1 inhibited it (Fig. 3c). Importantly, caspase-8 or BECLIN1 knock-down both impaired macrophage differentiation of monocytes as shown by a reduction of the double positive CD71/CD163 population that corresponds to differentiated macrophages (Fig. 3d). In conclusion, we show that the signaling pathways involved in monocyte differentiation by CSF-1 or IL-34 are strictly identical.

### CSF-1 and lL-34 macrophages exhibit differences in their ability to polarize into M1 or M2 macrophages

Globally, the results described above indicate that CSF-1 and lL-34-mediated intracellular signaling pathways, including auto-phosphorylation of the CSF-1R, activation of AKT, ERK 1/2, AMPK and ULK1 and induction of caspase activities and autophagy are similar in human primary monocytes induced to differentiate into macrophages, suggesting that the effects of lL-34 are mediated via the CSF1-R. As no obvious differences have been reported so far in the effects of CSF-1 and IL-34 on monocyte differentiation (Figs 1–3), we investigated the effects of both cytokines on macrophage polarization. Macrophages are categorized as “classically activated” pro-inflammatory M1-macrophages or an “alternatively activated” anti-inflammatory M2-macrophages^22^. They can differentiate into various activation states owing to the cytokine balance in their microenvironment. M1 phenotypic activation (pro-inflammatory), in response to interferon gamma (lFNγ) and lipopolysaccharide (LPS), is characterized by up-regulation of interleukin lL-6 and enhancement of the Th1 immune response. M2 phenotypic activation (anti-inflammatory) is stimulated by lL-4 and characterized by increased expression of lL-10^23^.

**Figure 4:**
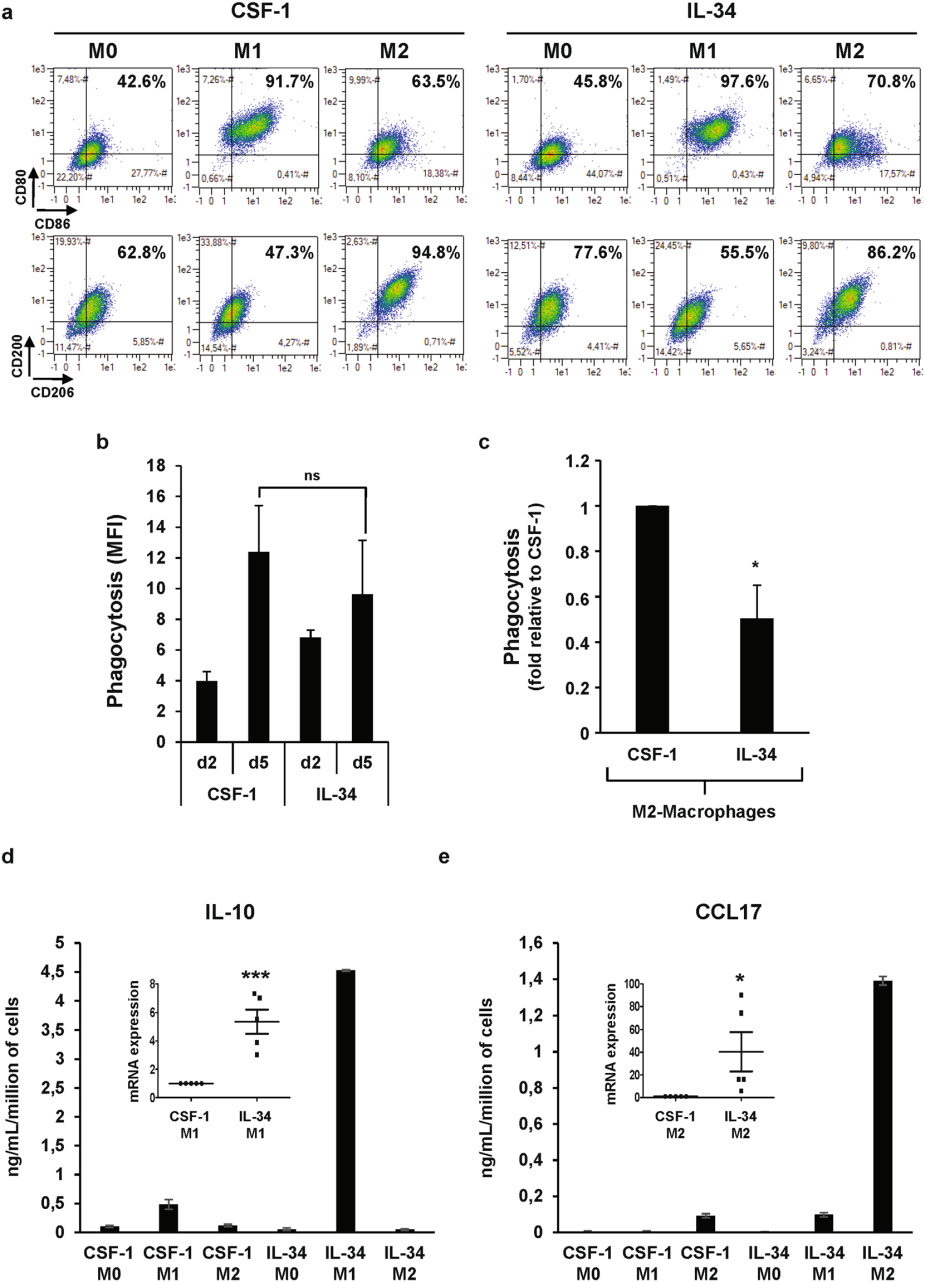
IL-34 macrophages have a different polarization potential as compared to CSF-1-macrophages. (**a**) Human monocytes were differentiated during 7 days with 100 ng/mL CSF-1 or 100 ng/mL IL-34 and then polarized into M1-macrophages (LPS + IFNγ) or M2-macrophages (IL-4) for 2 days. Macrophage polarization was evaluated by 2-color flow cytometric analysis. (**b**) Functional assay of monocytes exposed for 2 or 5 days to 100 ng/mL CSF-1 or 100 ng/mL IL-34. The results are expressed as MFI and represent the mean ± SD of 3 independent experiments performed in duplicate. n.s. denotes not statistically significant according to a paired student *t* test. (**c**) Functional assay of monocytes exposed for 7 days with 100 ng/mL CSF-1 or 100 ng/mL IL-34 and then polarized into M2-macrophages (IL-4) for 2 days. The results are expressed as the fold induction compared to CSF-1 macrophages and represent the mean of 3 independent experiments performed in duplicate. *P < 0.05 according to a paired student *t* test (versus CSF-1-macrophages). (**d**,**e**) Human monocytes were differentiated during 7 days with 100 ng/mL CSF-1 or 100 ng/mL IL-34 and then polarized into M1-macrophages (LPS + IFNγ) or M2-macrophages (IL-4) for 24 hours. The expression of the indicated mRNA  was analyzed by qPCR (mean ± SEM of 5 independent experiments). *P < 0.05, ***P < 0.001 according to a paired student *t* test (versus CSF-1 macrophages). The production of IL-10 and CCL17 was analyzed using Multi-Analyte ELISArray kit as described in Material and Methods section. The results are expressed as ng/mL per million of cells and represent the mean ± SD of 2 independent experiments performed in duplicate.

M1 or M2 polarization was initiated after 7 days of differentiation with either CSF-1 or lL-4 and next evaluated after a 2-day stimulation period with LPS + lNFγ or lL-4, respectively. ln a first series of experiments, the M1/M2 macrophage phenotype induced by lFNγ and LPS (M1 inducer) or IL-4 (M2 inducer) was analyzed by flow cytometry in macrophages differentiated with CSF-1 or lL-34. ln CSF-1 differentiated macrophages, LPS + lNFγ induced M1 polarization with both an increase in CD80 and CD86 expression and a decrease in CD200R and CD206 expression, whereas lL-4 promoted M2 polarization with an inhibition in CD80 and CD86 expression and an increase in CD200R and CD206 expression (Fig. 4a). Interestingly, whereas there was no difference in the ability of CSF-1 and lL-34 differentiated macrophages to phagocyte bacteria (Fig. 4b), M2-macrophages derived from CSF-1-stimulated monocytes exhibited a higher capacity to phagocyte bacteria than M2-macrophages derived from lL-34 differentiated macrophages (Fig. 4c).

To identify the cytokines that could be important for the polarization of macrophages in lL-34 stimulated monocytes, we analyzed by both real time RT-PCR and Elisa the expression level of some cytokines/chemokines known to be modulated during the M1 or M2 polarization process^24–26^. Regarding M1 polarization, we found a significant increase in IL-10 and CXCL11 mRNA and protein levels when IL-34 macrophages were polarized in response to LPS + IFNγ compared to CSF-1 polarized macrophages (Fig. 4d and Sup Fig. 3a). Some differences were also observed between macrophages polarized into the M2 phenotype by CSF-1 or IL-34. The most noticeable one were a very strong induction of CCL17, and CCL22, together with a decrease of IL-10 which was observed only when IL-34 differentiated monocytes were polarized into the M2 phenotype (Fig. 4e and Sup Fig. 3b). To investigate whether these differences in cytokine expression may be physiologically relevant, we analyzed the ability of M1 or M2 polarized macrophage secretomes (i.e from CSF-1 or IL-34 treated monocytes) to induce polarization of purified naïve T cells from two donors into the Th1 or Th2 phenotype. As no obvious differences were observed for Th2 polarization (not shown), we next focused on Th1 polarization. Therefore, we followed by flow cytometry the intracellular expression of IFNγ (Sup Fig. 3c and d) which is representative of Th1 cells. Of note, T cell polarization was higher in the presence of the secretome derived from M1-polarized CSF-1 macrophages as compared to M1-polarized IL-34 macrophages. Finally, the secretome derived from M2-polarized IL-34 macrophages strongly increased Th1 polarization compared to M2-polarized CSF-1 macrophages. In conclusion, although IL-34 and CSF-1 display an equivalent macrophage differentiation ability, the functionality of IL-34 and CSF-1 macrophages following polarization into the M1 or M2 phenotype is different, more specially regarding Th1 cell polarization.

## Discussion

CSF-1 and IL-34 share the same receptor^11^, the so-called CSF-1R and no obvious differences in the downstream signaling pathways triggered by both ligands in monocytes have been reported so far^27^. However, some genetic evidence indicate that it could exist differences in the effect of both cytokines in myeloid cells and in the brain^28,29^. This is exemplified by the fact that CSF-1 deficient (CSF-1^*op/op*^) mice exhibit a less severe phenotype than their CSF-1R deficient counterpart suggesting that at least one part of the IL-34 effect could be triggered independently of the CSF-1R^30^. This has been recently confirmed following the identification of a second receptor for IL-34 in the brain. Indeed, IL-34 binds to and signals by an alternative receptor, namely PTP-ζ^14^. Moreover, it has also been reported minor differences in the production and secretion of some cytokines including eotaxin-2 in peripheral blood cells stimulated with CSF-1 or IL-34^27^. Although CSF-1 and IL-34 exert many redundant functions *ex vivo*, the profile of expression, the source and the kinetic of cytokine production are different *in vivo*. Indeed, in contrast to CSF-1, IL-34 shows specific expression by keratinocytes and neurons in mice and plays an important role in the development and maintenance of Langherans cells and microglia, respectively^29,31^. In human, IL-34 is more widely/differently expressed, albeit also highly expressed in neuronal tissues and skin (see FANTOM5 and GTEX databases). There is also increasing evidence that IL-34 expression is upregulated in pathological conditions and may play important functions in autoimmune disorders, infections, inflammation and cancer^32^. Regarding cancer, IL-34 promotes the recruitment of M2-polarized tumor-associated macrophages by a direct effect on CSF-1R in macrophages, promoting new vessel formation and extravasation of immune cells, as CSF-1 does^33^. However, clear evidence indicating different functions of both cytokines are still lacking.

In the present study, we scrutinized the effect of CSF-1 and IL-34 on human monocyte differentiation. We found that the early and late signaling pathways leading to the differentiation of monocytes into macrophages were strictly identical in both conditions. Moreover, we also described for the first time that IL-34 promotes caspase activation and the activation of the AMPK/ULK1 pathway in monocytes leading to LC3-I lipidation and cleavage into LC3-II, as CSF-1 does, confirming the similarity of action of both ligands on human monocyte differentiation and induction of autophagy.

Whereas monocyte differentiation proceeded similarly in CSF-1 and IL-34 stimulated human primary monocytes, we identified however, differences in the production of some cytokines/chemokines when cells were polarized into the M1 or M2 phenotype using LPS + IFNγ or IL-4 following macrophage differentiation by either CSF-1 or IL-34. The most noticeable examples, were increased production of IL-10 and CXCL11 at the mRNA and protein level in M1 polarized macrophages. Regarding M2 polarization, we also observed a striking induction of CCL17 and CCL22 and a decrease in IL-10 mRNA and protein expression, which were found exclusively in M2-polarized IL-34 macrophages. Therefore, despite a strictly identical profile of monocyte differentiation, the cytokines/chemokines produced by CSF-1 or IL-34 macrophages are somehow different, suggesting that macrophages generated in both conditions may have different role in pro or anti-inflammatory pathophysiological conditions.

CCL17 (TARC) was originally implicated in the selective attraction of Th2 lymphocytes and thus considered as a M2 cytokine. Recent findings in the literature have reported that human monocyte-derived macrophages grown in CSF-1 do induce CCL17 in response to IL-4 as do mouse bone-marrow-derived macrophages grown in CSF-1^25,26^. It was also recently reported that GM-CSF drives CCL17 mRNA and protein expression in human and mice monocytes. Surprisingly, CCL17 seems to mediate at least some of the pro-inflammatory action of GM-CSF on arthritic pain and disease^34^. Several other cytokines including IL-4^35^ and IL-13^36^ have been reported to be associated with upregulation of CCL17 mRNA and protein. The fact that CCL17 mRNA expression was selectively upregulated in macrophages differentiated in the presence of IL-34 and polarized into the M2 phenotype by IL-4 but not in their CSF-1 differentiated counterpart indicates that polarization rather than IL-4 itself is responsible for CCL17 mRNA induction in our experiments. Globally, these findings indicate that IL-34 could resemble more GM-CSF than CSF-1 in its ability to trigger M2 polarization.

IL-10 is produced by Th2 lymphocytes and is known to suppress the differentiation and effector function of Th1^37–39^. We showed here, that IL-10 protein expression is strikingly increased in M1-polarized IL-34 macrophages compared to M1-polarized CSF-1 macrophages. This may explain the impairment of Th1 polarization observed with the secretome of M1-polarized IL-34 macrophages.

The reason for the differential effect of CSF-1 and IL-34 regarding the polarization into M1 or M2 phenotypes is currently unknown. However, a likely explanation could rely on the fact that IFNγR/TLR4 or IL-4 receptor expression is different in CSF-1 and IL-34 differentiated macrophages. We checked that it was not indeed the case (Sup Fig. 4). An alternative hypothesis would be that this specific effect of IL-34 is mediated independently of the CSF-1R. It has been described that IL-34 can bind CD138 and PTP-ζ that can act as putative IL-34 receptors. As monocytes and macrophages failed to express CD138 (syndecan 1) (Sup Fig. 4) and PTP-ζ expression seems restricted to neuronal tissues and skin, it would be interesting in future studies to investigate whether other proteoglycans such as serglycin, versican, perlecan or other syndecans^40,41^, including syndecan 4, could explain the differential effect of IL-34 regarding the polarization into M1 or M2 phenotypes.

In conclusion, the signaling pathways induced by CSF-1 and IL-34 downstream CSF-1R are undistinguishable. However, some differences in chemokines/cytokines production do exist when CSF-1 or IL-34 differentiated monocytes are induced to polarize into the M1 or M2 phenotypes following LPS + INFγ or IL-4 treatments. Variations in the secretome induced by CSF-1 or IL-34 may account for their different ability to polarize naïve T lymphocytes into Th1 cells. Strikingly, the increase in IL-10 production in M1-polarized IL-34 macrophages promoted a decrease in Th1 cell polarization while the increase in CCL17 and CCL22 in M2-polarized IL-34 macrophages favored an increase in Th1 cell polarization. These findings suggest that in an inflammatory context, IL-34 derived macrophages may behave differently than CSF-1 derived macrophages. Therefore, we conclude that IL-34 and CSF-1 display equivalent macrophage differentiation ability but different polarization potential.

## Material and Methods

### Reagents and antibodies

Human IL-34 was from Biolegend (BLE577906). Human CSF-1 was purchased from Miltenyi (130-096-493). Bafilomycin A1 was from Tocris (1334). qVD was from Clinisciences (A1901-5mg). 3-MethylAdenine was purchased from Sigma Aldrich (M9281). Phospho-CSF-1R (Tyr723), Phospho-AKT, AKT, Phospho-ERK1/2, ERK1/2, Phospho-AMPK (Thr172), AMPK, Phospho-ULK1 (Ser555), ULK1, Caspase-8, Caspase-3, Nucleophosmin, LC3-B, and Beclin antibodies were purchased from Cell Signaling Technology (catalog numbers were 3155, 4060, 9272, 4370, 4695, 2535, 2532, 5869, 8054, 9746, 9662, 3542, 2775, and 3738 respectively). CSF-1R, ACTIN, CD71, and CD163 antibodies were from Santa Cruz Biotechnology (catalog numbers were sc-692, sc-1616, sc-51829 and sc-33559 respectively). Cathepsin B was from MERCK (IM27L). HRP-conjugated rabbit anti-goat or mouse was purchased from Dako (P0449 and P0260) and HRP-conjugated goat anti-rabbit was from Cell Signaling (5127).

### Human monocyte culture and differentiation

Human peripheral volunteers were obtained from healthy donors with informed consent following the Declaration of Helsinki according to the recommendations of an independent scientific review board. The project has been validated by The Etablissement Français du Sang, the French national agency for blood collection (protocol N°ALM/PLER/FO/001). Blood samples were collected using ethylene diamine tetra-acetic acid–containing tubes. Mononucleated cells were first isolated using Ficoll Hypaque (Eurobio, CMSMSL0101). Then, we used the autoMACS® Pro Separator (Miltenyi, France) to perform cell enrichment. An initial positive selection, which included antibody targeting CD14, was used for monocyte enrichment (Miltenyi, 130-050-201). Purified monocytes from human were grown in RPMI 1640 medium with glutamax-I (Life Technologies, 61870044) supplemented with 10% (vol/vol) foetal bovine serum (Life Technologies). Monocytes were seeded in tissue culture treated flasks or wells. Macrophage differentiation was induced by adding into the culture medium 100 ng/mL CSF-1 or 100 ng/mL IL-34 and was visualized using standard optics (20x/0.35 Ph1) equipped with an AxioCam ERc camera (Zeiss, France). Phase images of the cultures were recorded with the Zen 2 software (Zeiss).

### Flow cytometry

To analyze the macrophagic differentiation of monocytes, cells were washed with ice-cold phosphate buffered saline (PBS, Life Technologies, 14190169), incubated at 4 °C for 10 min in PBS/bovine serum albumin (BSA 0.5%, Dutscher, 871002) with anti-CD71 and anti-CD163 or an isotype control (Miltenyi and BD Biosciences, catalog numbers were 551374 and 130-097-628). Finally, cells were washed and fixed in 2% paraformaldehyde (EMS, 15710). To detect caspase activity, we used FITC-DEVD-FMK according to the manufacturer’s instruction (Promokine, green caspase-3 staining kit, PK-CA577-K183). To perform macrophage polarization, purified monocytes were plated at 0.3 × 10^6^ per mL in RPMI 1640 medium with glutamax-I supplemented with 10% (vol/vol) fetal bovine serum plus CSF-1 or IL-34 for 7 + 2 days to differentiate into M0 macrophages. 100 ng/mL lipopolysaccharide (LPS-EK Ultrapure, Invivogen, tlrl-peklps) and 20 ng/mL interferon gamma (Miltenyi, 130-096-484) were added after 7 days of differentiation for two days to polarize into M1-macrophages. M2-macrophages were obtained by addition of 20 ng/mL IL-4 (Miltenyi, 130-094-117) for two days. To analyze the macrophage polarization, cells were detached using PBS/EDTA/BSA, washed with PBS, and incubated at 4 °C for 10 min in PBS/ bovine serum albumin with anti-CD80 (Miltenyi, 130-097-204), anti-CD86 (Miltenyi, 130-094-877), anti-CD200R (Biolegend, 329308), and anti-CD206 (Miltenyi, 130-100-034) or isotype controls. Finally, cells were washed and fixed in 2% paraformaldehyde (EMS, 15710). Fluorescence was measured with a MACSQuant® Analyzer (Miltenyi, Paris, France). To analyze the ability of macrophages to phagocyte bacteria, we used Vybrant® Phagocytosis Assay Kit according manufacture’s instruction (ThermoFisher, V-6694). Briefly, macrophages were detached and incubated with fluorescein-labeled E. coli (K-12 strain) for 30 min. Next, cells were washed twice with PBS and resuspended in PBS. Fluorescence, that indicate the internalization of particles, was measured with a MACSQuant® Analyzers (Miltenyi, France). Trypan blue solution was used to quench the fluorescence from particles that were not internalized.

### Immunoblot assays

Cells were lysed for 30 min at 4 °C in lysis buffer [50 mM HEPES pH 7.4, 150 mM NaCl, 20 mM EDTA, PhosphoSTOP (Sigma, 04906837001), complete protease inhibitor mixture (Sigma, 11836153001), 1% Triton X-100 (Sigma, T9284)]. Lysates were centrifuged at 20,000 g (15 min, 4 °C) and supernatants were supplemented with concentrated loading buffer (4X laemmli buffer). Fifty micrograms of proteins were separated and transferred following standard protocols before analysis with chemiluminescent detection kit (GE Healthcare, RPN2105).

### siRNA knockdown

Small interfering (si) RNAs were introduced into monocytes by nucleoporation (Amaxa, VPA-1007) of 5 × 10^6^ monocytes in 100 μL of nucleofector solution with 15 nmol of siRNA. Cells were incubated for 24 h with 5 mL of prewarmed complete medium, and CSF-1 or IL-34 was subsequently added. We used siRNAs (Life Technologies) targeting *Caspase-8* (HSS141461), *BECLIN1* (HSS112741) and *LUCIFERASE* as a negative control (Sense: 5′-CUUACGCUGAGUACUUCGAtt-3′).

### Reverse-transcription and real-time polymerase chain reaction

RNA was prepared from 5 × 10^6^ cells using the RNeasy Mini Kit according to manufacturer’s protocol (Qiagen, 74104). Each cDNA sample was prepared using AMV RT and random primers (Promega, M510F and C1181). Real-time polymerase chain reaction (PCR) was performed using the SyBR Green detection protocol (Life Technologies, 4367659). Briefly, 5 ng of total cDNA, 500 nM (each) primers, and 5 µL SyBR Green mixture were used in a total volume of 10 µL. Detection of multiple endogenous controls (*ACTB, L32 and UBIQUITIN*) were used to normalize the results. Specific forward and reverse primers are accessible upon request.

### Cytokine production assay

To perform macrophage polarization, purified monocytes were plated at 0.3 × 10^6^ per mL in RPMI 1640 medium with glutamax-I supplemented with 10% (vol/vol) fetal bovine serum plus CSF-1 or IL-34 for 7 + 1 days to differentiate into M0 macrophages. 100 ng/mL lipopolysaccharide (LPS-EK Ultrapure, Invivogen, tlrl-peklps) and 20 ng/mL interferon gamma (Miltenyi, 130-096-484) were added after 7 days of differentiation for one day to polarize into M1-macrophages. M2-macrophages were obtained by addition of 20 ng/mL IL-4 (Miltenyi, 130-094-117) for one day. Next, cells were washed and seeded in RPMI 1640 medium with glutamax-I supplemented with 10% (vol/vol) fetal bovine serum during one day. The cytokines productions were finally evaluated using MultiAnalyte ELISArray kit (Qiagen, MEH-004A and MEH-009A).

### Statistical analysis

Statistical analysis was performed using a paired Student *t* test and significance was considered when *P* values were lower than 0.05. The results are expressed as the mean ± SEM.

## Electronic supplementary material

Supplementary information
