# Supplementary material for: IL-34 and CSF-1 display an equivalent macrophage differentiation ability but a different polarization potential
Source: Sci Rep. 2018 Jan 10;8:256. doi: 10.1038/s41598-017-18433-4 (PMC5762882; doi:10.1038/s41598-017-18433-4)
Supplement: Supplementary file 1 — Supplementary information [file 41598_2017_18433_MOESM1_ESM.pdf]

IL-34 and CSF-1 display an equivalent macrophage differentiation ability but a different polarization potential

Sonia Boulakirba[1], Anja Pfeifer[1], Rana Mhaidly[1], Sandrine Obba[1], Michael Goulard[1], Thomas Schmitt[1], Paul Chaintreuil[1], Anne Calleja[1], Nathan Furstoss[1], François Orange[2], Sandra Lacas-Gervais[2], Laurent Boyer[1], Sandrine Marchetti[1], Els Verhoeyen[1], Frederic Luciano[1], Guillaume Robert[1], Patrick Auberger[1#*] and Arnaud Jacquel[1#*]

1- Université Côte d'Azur, C3M Inserm U1065, Nice, France

2- Université Côte d'Azur, Centre Commun de Microscopie Appliquée, Nice, France

* Corresponding author

# Equal contribution

Correspondence:

Dr. Jacquel Arnaud / Dr. Auberger Patrick

Centre Méditerranéen de Médecine Moléculaire (C3M)

Inserm U1065, Bâtiment ARCHIMED, 151, route de Saint-Antoine de Ginestière

BP2 3194, 06204 Nice cedex 03, France

Phone: +33 4 89 06 43 06

Email: jacquel@unice.fr

Key words: primary monocyte, macrophage, differentiation, IL-34, CSF-1, autophagy, caspase, polarization

Supplemental material and methods

Electronic microscopy - Monocyte/Macrophage cell pellets were collected, fixed with 1.6% glutaraldehyde, post fixed in 1% OsO4, dehydrated in alcohol series, and embedded in epoxy resin. Thin sections were contrasted with uranyl acetate and lead citrate. Samples were analyzed with JEOL 1200EX electron microscope.

Flow cytometry - To analyze the expression of IFN-γ receptor (CD119), Toll-like receptor 4 (CD284), IL-4R (CD124) and CD138, cells were washed with ice-cold PBS, incubated at 4°C for 10 min in PBS/BSA with anti-CD119 (Miltenyi, 130-099-920), anti-CD284 (Miltenyi, 130-100-378), anti-CD138 (Miltenyi, 130-091-250) and anti-CD124 (Biolegend, 355003) antibodies or isotype controls. Cells were washed and fixed in 2% paraformaldehyde (EMS, 15710). Fluorescence was measured with a MACSQuant® Analyzer (Miltenyi, Paris, France).

T cell polarization assays – Naïve T lymphocytes were purified from 2 healthy donors using the Naive CD4+ T Cell Isolation Kit II (Miltenyi, 130-094-131). Secretomes from M0, M1 or M2 polarized macrophages differentiated with either 100 ng/mL CSF-1 or IL-34, were used to induce polarization of $1x10^6$ naïve T cells per mL during 12 days. For detection of intracellular cytokines, T cells were stimulated for 5 h with 1 µg/mL GolgiPlug containing brefeldin A (BD Pharmingen, 555029) + 50 ng/mL PMA (Sigma, P8139) + 500 ng/mL ionomycin (Sigma, I0634) . Cells were harvested and stained with anti-CD4 (Miltenyi, 130-114-725). Cells were next washed, fixed with 70 µL Cytofix (BD Pharmingen, 554714) and incubated for 20 min at 4˚C. Cells were then washed with Perm/Wash buffer (BD Pharmingen) and permeabilized with 70 µL cytoperm during 20 min at 4°C. Intracellular Abs for IL-4 (Miltenyi, 130-098-815) and IFNγ (Miltenyi, 130-097-944) were added in 50 µL Perm/Wash buffer. After a 30 min incubation at 4˚C, cells were washed twice with PBS and fluorescence was measured with a MACSQuant® Analyzer (Miltenyi, Paris, France).

Supplemental legends

Supplemental Figure 1. IL-34 or CSF-1 treatment lead to an equivalent accumulation of specific membrane markers such as CD71 or CD163. Human peripheral blood monocytes from healthy donors were exposed to 100 ng/mL CSF-1 or 100 ng/mL IL-34 for the indicated times. Macrophage differentiation of monocytes from 3 different healthy donors was followed by flow cytometry analysis. The percentage indicates cells that express CD71 (a) or CD163 (b). (c) Human peripheral blood monocytes from healthy donor were exposed to the indicated concentrations of CSF-1 or IL-34 during one or two days. Macrophagic differentiation of monocytes was followed by 2-color flow cytometric analysis. The percentage indicates cells that express both CD71 and CD163. Each panel is representative of at least 3 independent experiments.

Supplemental Figure 2. IL-34 or CSF-1 stimulation leads to an autophagy activation. (a) Human peripheral blood monocytes from healthy donors were exposed to 100 ng/mL CSF-1 or 100 ng/mL IL-34 for the indicated times. Electron microscopy images showing ultrastructural features of a representative monocyte (d0) and morphologic features of autophagy in monocytes treated for 3 days (d3) with CSF-1 or IL-34. A indicates Autophagosome and N, nuclei. (b) Monocytes were exposed for 3 days to 100 ng/mL CSF-1 or 100 ng/mL IL-34 alone or in association with bafilomycin A1 (15 nM) added 3 hours after CSF-1 or IL-34 treatment. Protein expression was analysed by immunoblot. Actin is used as a loading control. Asterisk indicates a cleavage fragment. The ratio of the LC3-II protein level to that of LC3-I protein level was determined using ImageJ software. Each panel is representative of at least 3 independent experiments. (c) Human peripheral blood monocytes from healthy donors were exposed to the indicated concentrations of CSF-1 or IL-34 for 10 min. Immunoblot analysis of signaling proteins in monocytes following CSF-1 or IL-34 stimulation. P indicate phosphorylated proteins. Each panel is representative of at least 3 independent experiments.

Supplemental Figure 3. IL-34 macrophages have a different polarization potential compared to CSF-1-macrophages. (a-b) Human monocytes were differentiated during 7 days with 100 ng/mL CSF-1 or 100 ng/mL IL-34 and then polarized into M1-macrophages (LPS+IFNγ) or M2-macrophages (IL-4) for 24 hours. The expression of the indicated mRNA is analyzed by qPCR (mean ±SEM of 5 independent experiments). $^{*}P < 0.05$, $^{**}P<0.01$ according to a paired student *t* test (versus CSF-1-macrophages). (c-d) Secretomes from M0, M1 or M2 polarized macrophages differentiated with either 100 ng/mL CSF-1 or IL-34 were used to induce polarization of naïve T cells during 12 days. This polarization was performed with two different donors (c and d). Th1 polarization was examined by 2-color flow cytometric analysis. The percentage indicates cells that express both CD4 and IFNγ which corresponds to Th1 cells.

Supplemental Figure 4. IL-34 or CSF-1 stimulation leads to the identical expression of IFNγR, TLR4 and IL-4 receptor. Human monocytes were differentiated during 7 days with 100 ng/mL CSF-1 or 100 ng/mL IL-34. The expression of IFN-γ receptor (IFNR), Toll-like receptor 4 (TLR4), IL-4R and CD138 was analyzed by flow cytometry.

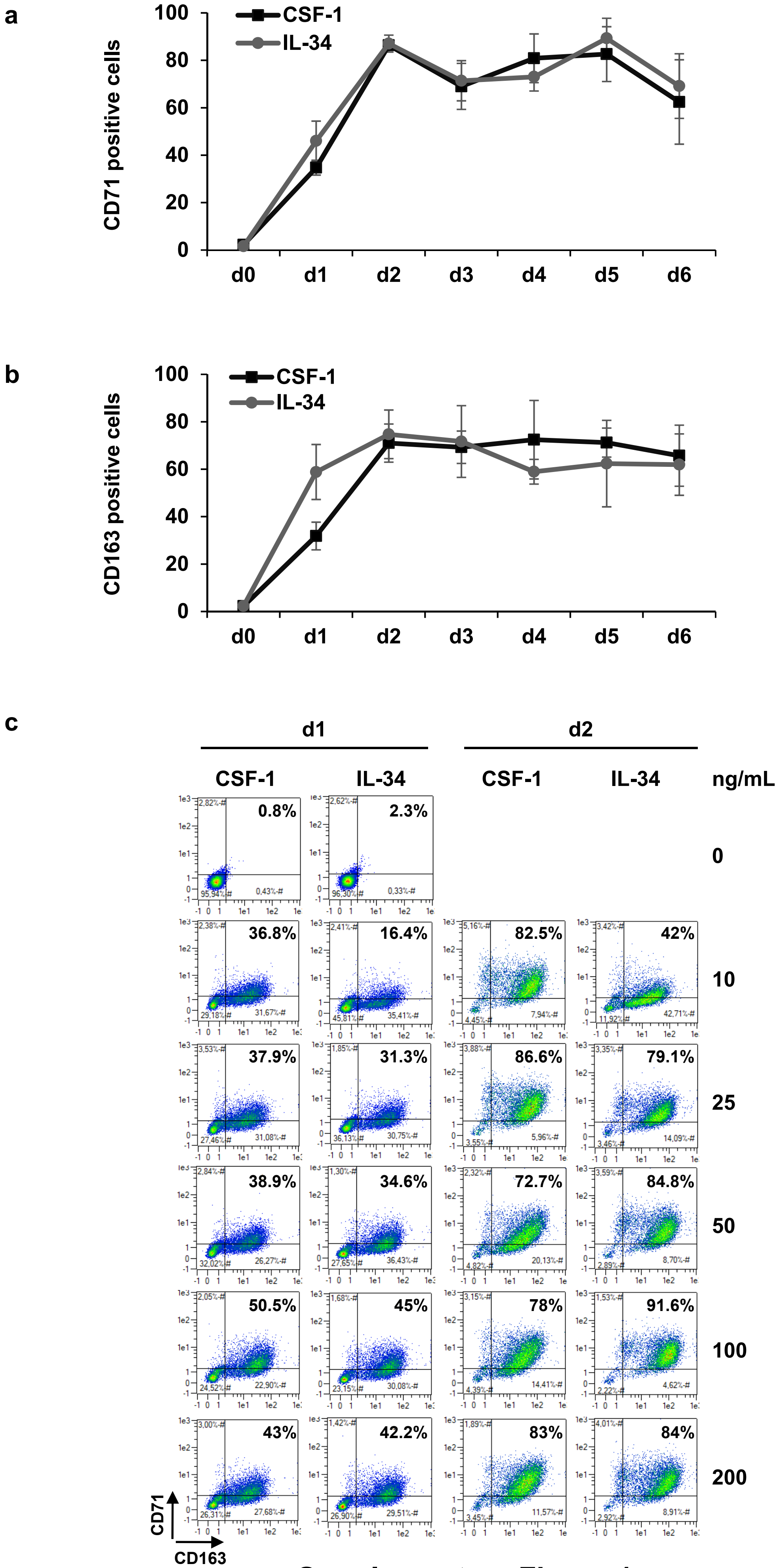

Supplementary Figure 1

Boulakirba et al.

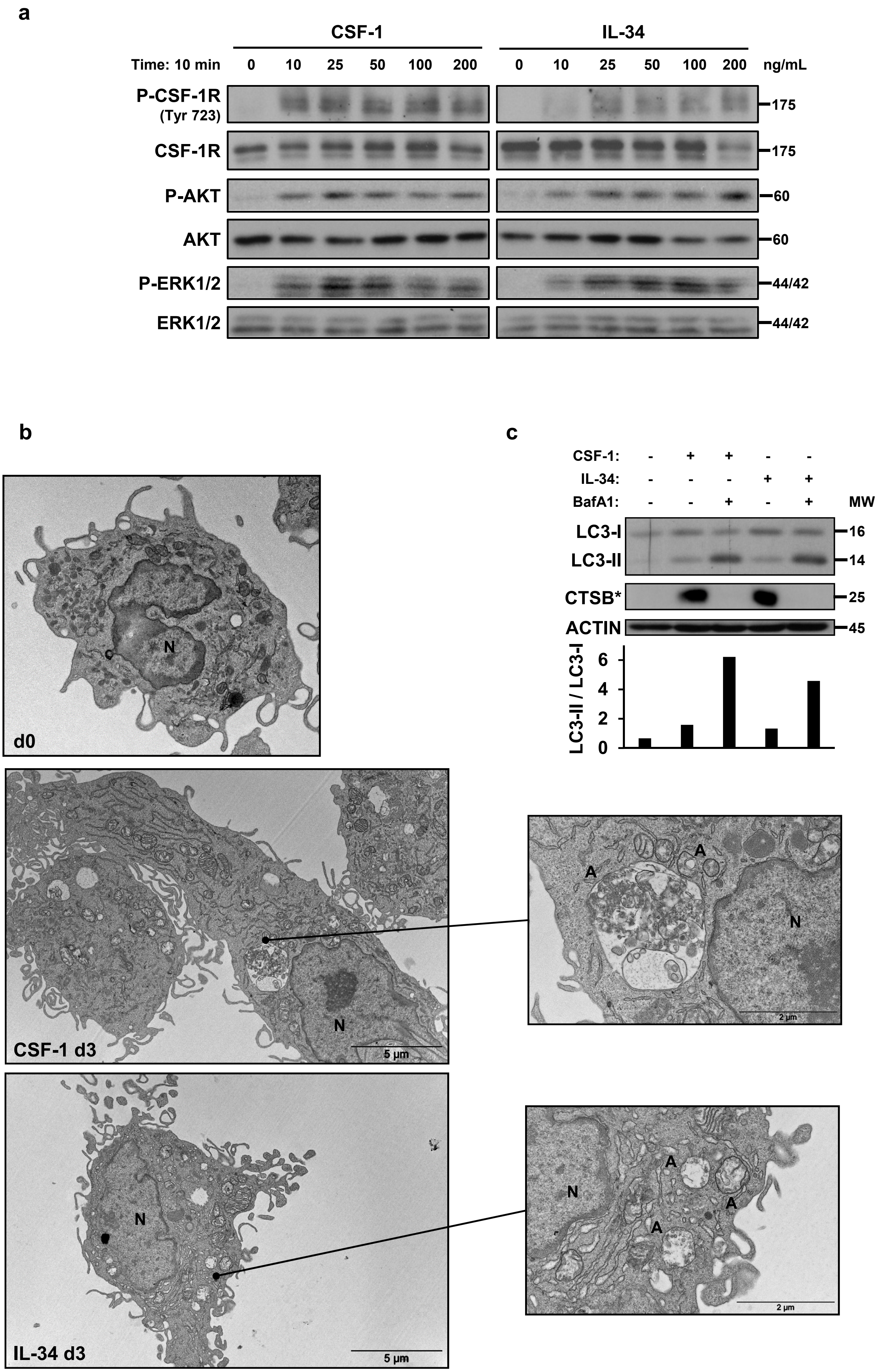

Supplementary Figure 2

Boulakirba et al.

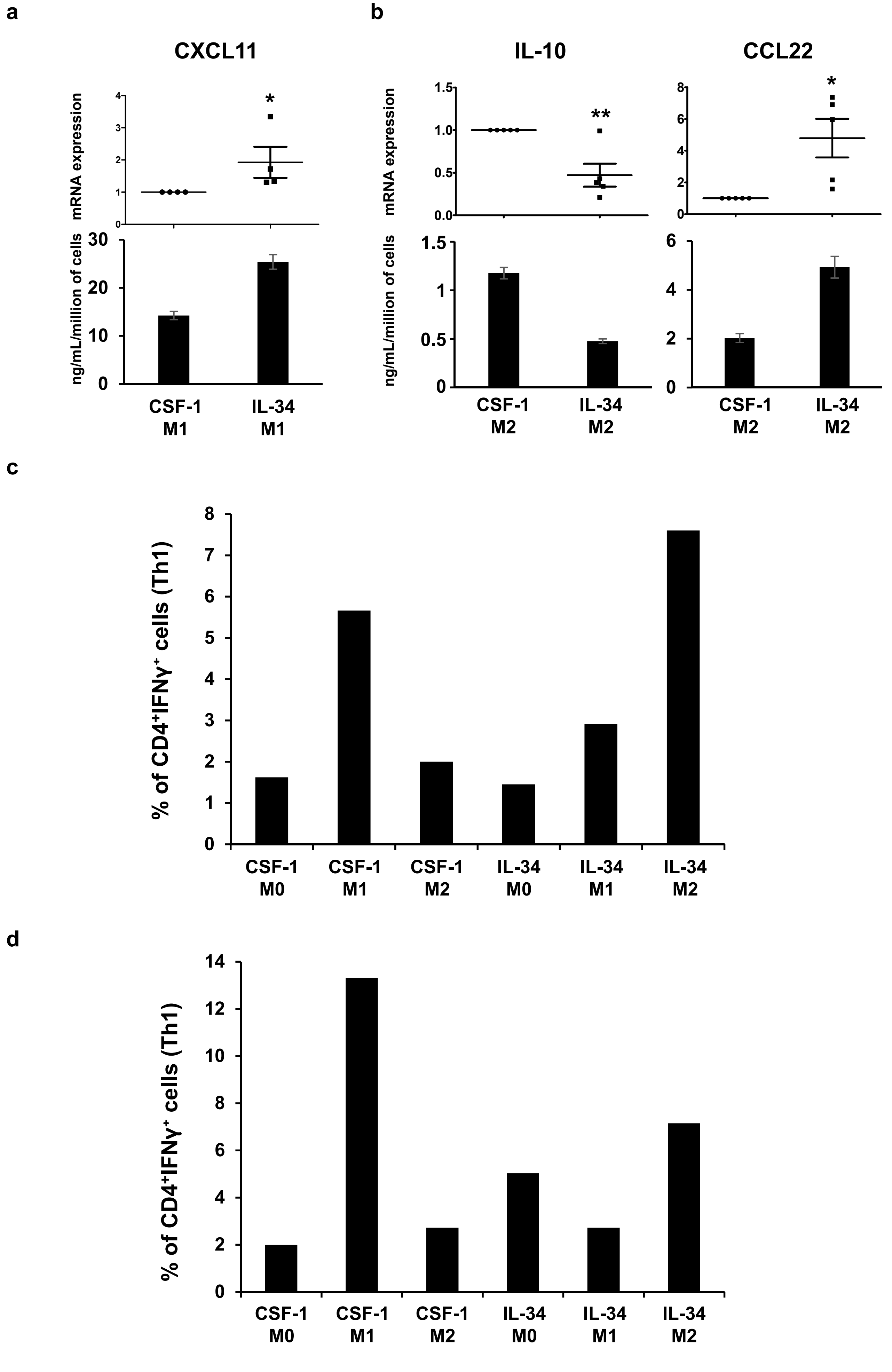

Supplementary Figure 3

Boulakirba et al.

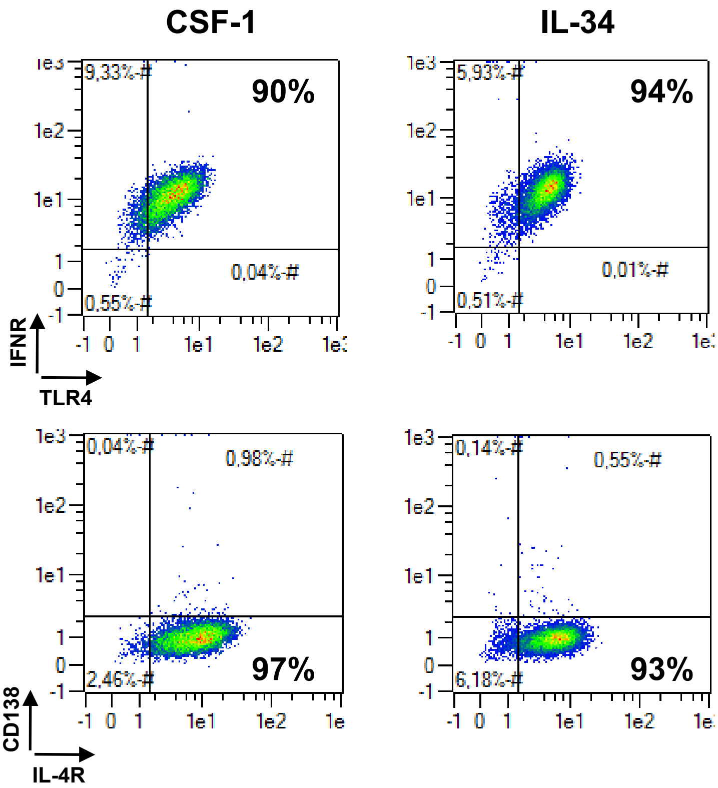

Supplementary Figure 4 ***Boulakirba et al.***
